# Identification of EMT-associated prognostic features among grade II/III gliomas

**DOI:** 10.1038/s41598-024-53399-0

**Published:** 2024-02-03

**Authors:** Wenyong Yang, Liangbin Lin, Tianqi Lu, Hui Yu, Sunfu Zhang

**Affiliations:** 1 Department of Neurosurgery, Department of Urology, Medical Research Center, The Third People’s Hospital of Chengdu, The Affiliated Hospital of Southwest Jiaotong University, The Second Chengdu Hospital Affiliated to Chongqing Medical University, Chengdu, China; 2 The Center of Gastrointestinal and Minimally Invasive Surgery, Department of General Surgery, The Third People’s Hospital of Chengdu, The Affiliated Hospital of Southwest Jiaotong University, Chengdu, China; 3 Obesity and Metabolism Medicine-Engineering Integration Laboratory, Department of General Surgery, The Third People’s Hospital of Chengdu, The Affiliated Hospital of Southwest Jiaotong University, Chengdu, Sichuan China

**Keywords:** CNS cancer, Tumour biomarkers

## Abstract

Grade II/III gliomas have a highly heterogeneous clinical course. Identifying prognostic biomarkers in grade II/III gliomas is essential to guide clinical management. We explored epithelial-mesenchymal transition (EMT)-related genes to uncover prognostic features in grade II/III gliomas. Consensus cluster analysis of 200 EMT-related genes classified 512 grade II/III glioma samples into two molecular subtypes, C1 and C2. The C1 subtype had significantly worse overall survival compared to the C2 subtype. Pathway analysis revealed C1 tumors were highly associated with tumor progression pathways and demonstrated higher immune cell infiltration scores. Differential expression analysis identified four genes (ACTN1, AQP1, LAMC3, NRM) that discriminated the two subtypes. Validation in external datasets confirmed that high expression of this four-gene signature predicted poor prognosis in grade II/III gliomas. Cellular experiments showed ACTN1, AQP1 and NRM promoted glioma cell proliferation, migration and invasion. We examined correlations of the signature genes with T cell exhaustion markers and found ACTN1 expression had the strongest association. Immunohistochemistry analysis further demonstrated that ACTN1 protein expression in grade II/III gliomas was negatively correlated with patient overall survival. In summary, our study identified a concise four-gene signature that robustly predicts grade II/III gliomas prognosis across multiple datasets. The signature provides clinical relevance in distinguishing more aggressive grade II/III glioma tumors. Targeting the ACTN1, AQP1 and NRM genes may offer new therapeutic opportunities to improve grade II/III gliomas patient outcomes.

## Introduction

Glioma is the most common malignant primary brain tumor in adults, and it is also one of the most common fatal tumors, with an increasing incidence year by year^1^. The number of newly diagnosed glioma cases worldwide each year is approximately 1.6 million, with an age-standardized incidence rate of about 19 per 100,000 population^2^. Currently, the preferred treatment for glioma is maximally safe resection, followed by postoperative radiotherapy combined with temozolomide or nitrosamine chemotherapy^3^. Despite the continuous development of treatment methods in recent years, the prognosis of glioma is still poor due to the difficulty of complete resection by surgery, the toxic side effects and drug resistance of traditional chemotherapy, and the median survival time of glioma is only 15–17 months^4^. Tumor immunotherapy is regarded as another milestone new tumor treatment strategy after surgery, radiotherapy, chemotherapy, and targeted therapy. Therefore, further in-depth study of the immune microenvironment of glioma helps develop new immunotherapy regimens, which is of great significance in improving the survival rate and quality of life of glioma patients^5^. The grade II/III gliomas have a slower course and are lowly aggressive compared to glioblastoma (GBM, grade IV). Therefore, classifying grade II/III gliomas and identifying the prognostic markers can provide a molecular basis for the precise treatment of gliomas.

This study focused on grade II/III gliomas, which are WHO grade II/III gliomas^6^. The grade II/III gliomas have a relatively better prognosis than GBM, accounting for about one-third of all central nervous system tumors^7^. With the improvement of the understanding of grade II/III gliomas, it is not only a tumor tissue but also a chronic central nervous system disease with aggressive and progressive nature. The grade II/III glioma cells can migrate with the white matter and eventually develop into a highly malignant GBM^8^. In recent years, numerous studies have revealed the complicated molecular mechanisms of glioma occurrence, including epithelial-mesenchymal transition (EMT), tumor-mesenchymal interaction, tumor microenvironment, tumor cell dormancy, and tumor stem cells^9^. A comprehensive understanding of these mechanisms may facilitate the development of novel therapeutic strategies and improve the prognosis of patients.

EMT is an important biological event in embryonic development, wound healing, cancer progression, and so on^10^. The essence of EMT is the developmental process by which quiescent cells acquire migration ability^11^. In the context of cancer, the EMT process is frequently reactivated, which leads to an increased invasive and metastatic capacity of cancer cells^12,13^. There is increasing evidence that EMT can make tumors have stem cell-like characteristics, leading to drug resistance and tumor relapse. Therefore, EMT is considered one of the central mechanisms determining the invasive potential cancer cells^11^. Similarly, EMT forms the immunosuppressive microenvironment, resulting in impaired antitumor immune function. For example, EMT can destroy immune synapses and ultimately impair T cell CD8-mediated cellular immune function^14^. In addition, EMT can up-regulate the expression of immune checkpoints, which are associated with immune escape and decreased sensitivity to immunotherapy^15^. Substantial evidence suggests that EMT is linked to the progression of various tumor types, including grade II/III gliomas^16^. Although the underlying mechanisms of EMT in grade II/III gliomas have been widely studied, the biological role and prognostic value of EMT-related genes remain elusive. Therefore, it is of great significance to analyze the molecular subtypes of grade II/III gliomas associated with EMT and evaluate their prognostic value to screen therapeutic targets and facilitate the clinical outcome of grade II/III glioma patients.

The grade II/III gliomas are the most common type of primary brain tumors in adults. EMT has been implicated as an important mechanism promoting invasion and metastasis in many cancer types, including gliomas. However, the role of EMT in grade II/III gliomas prognosis and pathogenesis remains poorly understood. In this study, our central hypothesis is that EMT-associated gene expression patterns can be used to define molecular subtypes of grade II/III gliomas that correlate with clinically relevant phenotypes. We constructed molecular subtypes of the grade II/III gliomas model based on EMT-associated genes. We assessed the correlation between molecular subtypes and the prognostic and clinical features of grade II/III glioma patients. A four-gene signature (ACTN1, AQP1, LAMC3, NRM) prognostic risk model was constructed among the differentially expressed genes (DEGs) identified in the grade II/III gliomas molecular subtypes. Furthermore, we validated the predicted prognosis value of our four-gene signature by using the Chinese Glioma Genome Atlas (CGGA) gene expression dataset. Taken together, our four-gene prognostic signature exhibits good performance for predicting the prognosis of grade II/III glioma patients and provides promising therapeutic targets for grade II/III gliomas treatment.

## Materials and methods

### Data sources and preprocessing

RNA-seq data and clinical information from grade II/III gliomas patient tissues were obtained from The Cancer Genome Atlas (TCGA). The data processing step was as follows: (1) samples without clinical information were deleted; (2) sets were transformed into gene symbols; (3) take the maximum value when multiple Gene Symbol expressions exist.

The grade II/III gliomas test RNA-seq data were obtained from the CGGA database, and data processing steps were as follows: samples without clinical information were deleted.

After preprocessing the three data sets, 512 samples were downloaded from TCGA-LGG; 159 samples were downloaded from CGCA-mRNA-array_301; 172 samples were obtained from CGCA-mRNAseq_325; 420 samples were obtained from CGCA-mRNAseq_693.

Genes related to EMT pathways (hallmark_epithelial al_MESENCHYMAL_TRANSITION) were obtained from MSigDB (Molecular Signature Database v7.0). There are A total of 200 EMT-related genes.

### The ConsensusClusterPlus algorithm was used to identify molecular subtypes

TCGA mRNA expression profile data were first filtered to remove genes with an expression level of less than 1, representing less than 50% of all samples. Then univariate COX analysis was then performed under a threshold of *P* < 0.05 to filter out unnecessary genes. The EMT genes related to prognosis were obtained, and then ConsensusClusterPlus was used to analyze consensus clustering on TCGA samples (V1.48.0; Parameters: reps = 100, pFeature = 1, pItem = 0.8, distance = "spearman ").

Euclidean distance and D2 were used as the distance metric and clustering algorithm to obtain 2 molecular subtypes. The limma package was used to analyze differences between molecular subtypes and to perform functional enrichment analysis. DAVID was used in the grade II/III gliomas dataset to calculate significantly enriched pathways in the two groups, including GO and KEGG pathways^17^. The GSEA input files containing TCGA mRNA expression profile data and molecular subtypes were labeled as group C1 or group C2 according to the sample label. The enrichment path was selected based on a *P* < 0.05 threshold and FDR < 0.25.

### Construction of prognostic risk model based on EMT genes

Accurate assessment of prognosis is critical for guiding treatment decisions and improving outcomes for cancer patients. Currently, prognostic estimates rely heavily on traditional clinical and pathological factors, which have modest accuracy. Molecular signatures that capture tumor heterogeneity can markedly strengthen prognostic prediction and enable personalized risk stratification. First, we obtained the expression data of EMT genes from a publicly available dataset. Then, we used univariate Cox regression analysis to identify EMT genes that were significantly associated with survival outcomes. Next, we performed Lasso regression analysis to further narrow down the number of genes and avoid overfitting of the model. The selected genes were used to construct a prognostic risk model using multivariate Cox regression analysis. The risk score was calculated for each patient based on the expression levels of the selected genes and their corresponding coefficients in the model. Patients were then divided into high-risk and low-risk groups based on the median risk score. Finally, we assessed the predictive performance of the risk model using receiver operating characteristic (ROC) analysis and Kaplan–Meier survival analysis. The construction of the prognostic risk model based on EMT genes aimed to provide a more accurate prediction of survival outcomes for cancer patients and guide personalized treatment decisions.

### Partitioning of training and validation sets

The 512 samples in the TCGA dataset were randomly divided into a training set and a test set according to the ratio of the training set: test set = 1:1. The most appropriate training and test sets were selected according to the following setting conditions: (1) the age distribution, gender, follow-up time, and mortality rate of patients in the two groups were similar; (2) after gene expression profiling clustering, the number of binary samples between randomly assigned groups was similar. This selection yielded 256 samples in the training and 256 in the test sets.

### Lasso cox regression analysis

To reduce the number of genes (1864 DEGs) in the RiskScore model, Lasso regression was applied to prognostic genes. Lasso regression can shrink parameters by setting partial coefficients. This biased estimation deals with the multicollinearity of the data, and it implements parameter estimation, problem-solving capabilities, and variable selection of multicollinearity in regression analysis. The motion trajectories of the respective variables were analyzed by lasso cox regression via the R package glmnet. Next, the optimal model was constructed by five-fold cross-validation, and then analyzed the confidence intervals under each lambda were to determine the number of target genes.

### Cell transfection

Small interfering RNA against negative control (NC-siRNA), ACTN1 (ACTN1-siRNA), LAMC3 (LAMC3-siRNA), AQP1 (AQP1-siRNA) and NRM (NRM-siRNA) were all synthesized by GenePharma (Shanghai, China). The sequences were as follows: si ACTN1-Homo-2036, 5′-CAGGAGAUCAAUGGCAAAUTT-3′; si-ACTN1-Homo-2421, 5′-GGACCAUCAAUGAGGUAGATT-3′; si AQP1-Homo-333, 5′-GUGCCCUCAUGUACAUCAUTT-3′; si AQP1-Homo-60, 5′-CCAGCGAGUUCAAGAAGAATT-3′; si LAMC3-Homo-1136, 5′-GCUGUCAGGAGAAUUUCUATT-3′; si LAMC3-Homo-1405, 5′-GGCAACCUAUGUGACAGAUTT-3′; si NRM-Homo-796, 5′-GGCUCCUCAUCUUUAGCAUTT-3′; si NRM-Homo-706, 5′-CAUACCCAAAGGCCCUGUGTT-3′; Cell transfection was conducted by using Hieff Trans Liposomal Transfection Reagent (Yeasen Biotechnology (Shanghai)) according to the manufacturer’s protocol.

### MTT and CCK-8 assays

Cell proliferation assays were performed using SW1088 and SW1733 cells in a 96-well plate, which are gift from Dr. Jingwen Jiang’s lab. The cells were seeded at a density of 3000 cells/well in 100 µl of 10% FBS DMEM. For the Cell Counting Kit-8 (CCK-8) assays, the original medium in each group was replaced with 10 µl of CCK-8 solution diluted in 100 µl of complete culture medium, following the protocol provided by Biosharp. After an additional 1-h incubation in the dark at 37 °C, the viable cells were detected by measuring the absorbance at a wavelength of 450 nm. For the MTT assays, 10 µl of MTT solution (Solarbio) was added to each well after 48 h of cell culture. After a 2-h incubation, the medium was discarded, and the optical density was measured at 490 nm using a Microplate Reader.

### Wound healing assay

The SW1088 cells were seeded in a six-well plate until a confluent cell monolayer was formed. To create a scratch, a 200-µl plastic pipette tip was gently used. The cells were then washed three times with PBS to remove any cell debris. Wound photographs were captured at 48 h after scratch using an IX71 inverted microscope from Leica Corporation and analyzed using ImageJ software (v1.80). The wound closure was measured by analyzing the wound area in ImageJ software. Specifically, the boundaries of the wound were outlined manually and the area was quantified at 0 h and 48 h after scratching. The percentage of wound closure was then calculated using the formula:

$$\% {\text{ wound closure }} = \, \left[ {\left( {{\text{Area}}0{\text{hrs }} - {\text{ Area48hrs}}} \right)/{\text{Area}}0{\text{hrs}}} \right] \, \times { 1}00$$

All experiments were performed in triplicate as per our study protocol.

### Transwell assay

The transwell assay was performed using Matrigel-coated transwell chambers. Transfected cell lines were cultured and then added to the upper chamber filled with serum-free medium, while the lower chamber contained complete culture medium. After incubation, non-invading cells in the upper chamber were removed, and invaded cells in the lower chamber were fixed and stained. Cell invasion was quantified by counting the number of invaded cells in ten random fields. This assay allows for the assessment of cell invasion capabilities and the evaluation of the impact of specific genes or experimental conditions on cell invasiveness.

### Immunohistochemistry

Formalin-fixed paraffin-embedded grade II/III glioma sections were deparaffinized, rehydrated, and subjected to heat-induced antigen retrieval. Endogenous peroxidase activity was blocked prior to overnight incubation at 4 °C with anti-ACTN1 primary antibody (1:200 dilution, servicebio). Signal was detected using an HRP-conjugated secondary antibody and DAB substrate. Sections were counterstained with hematoxylin. Stained slides were assessed by light microscopy.

### Ethics approval and consent to participate

The human low grade glioma tissue microarray utilized for ACTN1 immunohistochemistry analysis collected at The Third People's Hospital of Chengdu, the Second Chengdu Hospital Affiliated to Chongqing Medical University, under an approved protocol by the Institutional Ethics Board. The studies were conducted in accordance with the International Ethical Guidelines for Biomedical Research Involving Human Subjects (CIOMS), and the research protocols were approved by the Ethics Committee of The Third People's Hospital of Chengdu, the Second Chengdu Hospital Affiliated to Chongqing Medical University. All samples were obtained with informed consent.

## Results

### Molecular subtyping based on a non-negative matrix factorization (NMF) algorithm

**Figure 1:**
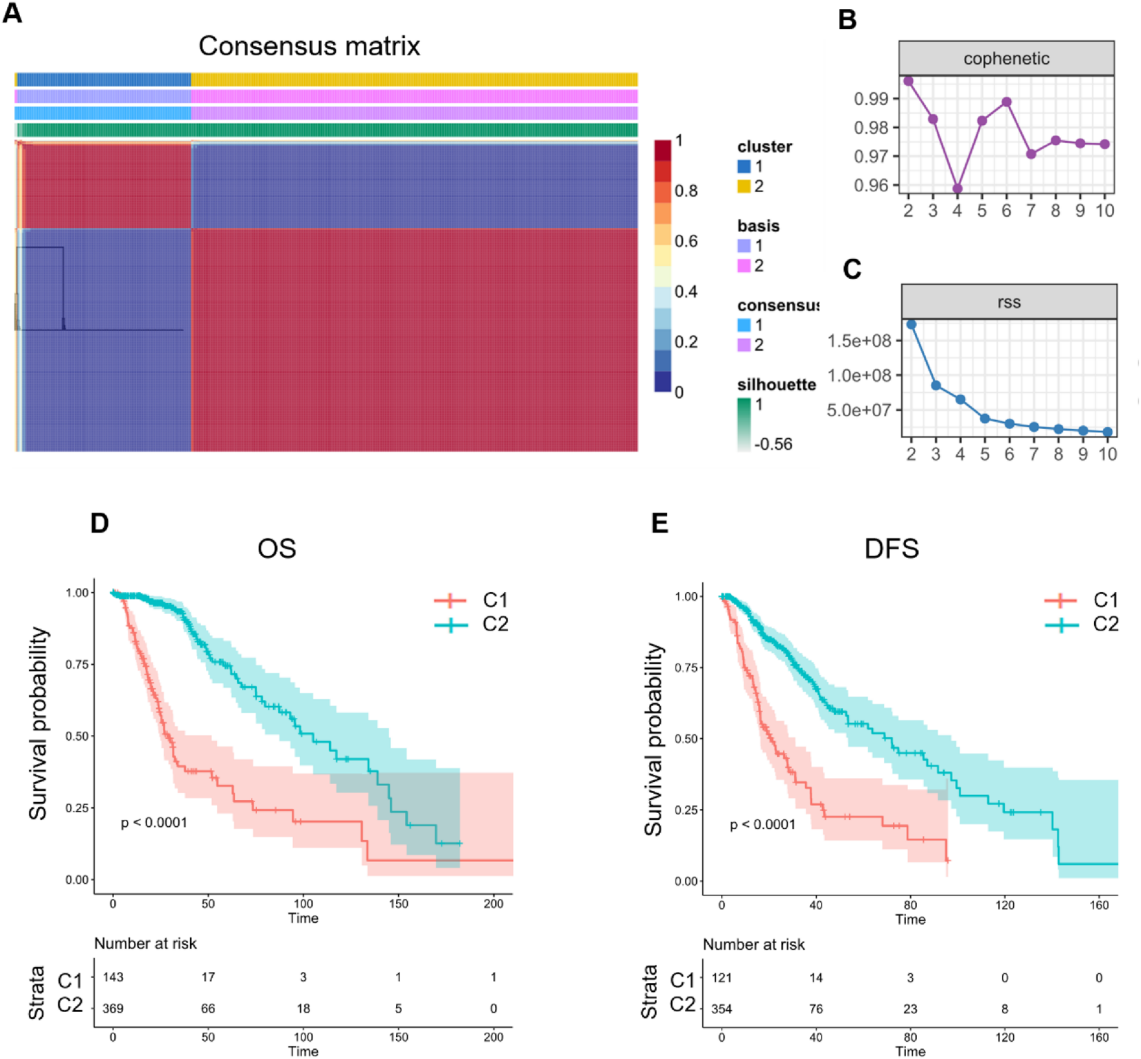
Molecular subtyping and prognostic analysis in grade II/III gliomas based on NMF clustering. (**A**) Consensus matrix plot of NMF clustering. (**B**) Cophenetic distribution with rank = 2–10, where the cophenetic correlation is derived from the consistency matrix proposed by Brunet et al., reflects the stability of the clusters obtained by NMF. This value ranges from 0 to 1, with higher values indicating more stable clusters. (**C**) RSS distribution, rank = 2–10. RSS is the residual sum of the sum of squares and represents the clustering performance of the model, where the smaller the value is, the better the clustering effect of the model is. (**D**) Overall survival (OS) prognostic survival curve of molecular subtypes. (**E**) DFS prognostic survival curve of molecular subtypes.

The expression profiles of 200 EMT-associated genes were first extracted from TCGA LGG data. Then univariate cox analysis with the coxph function in R yielded 132 genes related to grade II/III gliomas prognosis (Table S1) (*P* < 0.05). Based on the expression of 132 genes, we clustered the grade II/III glioma samples by NMF. The optimal clustering of k = 2 was selected based on the synthesis, residual sum of squares, and other metrics, resulting in 2 molecular subtypes. We named them C1 and C2 (Fig. 1A–C).

Further analysis revealed significant differences in overall survival (OS) time and disease-free status (DFS) time between groups C1 and C2. As shown in Fig. 1D,E, subtype C1 has a poor prognosis.

### Comparative analysis of immune scores among molecular subtypes

**Figure 2:**
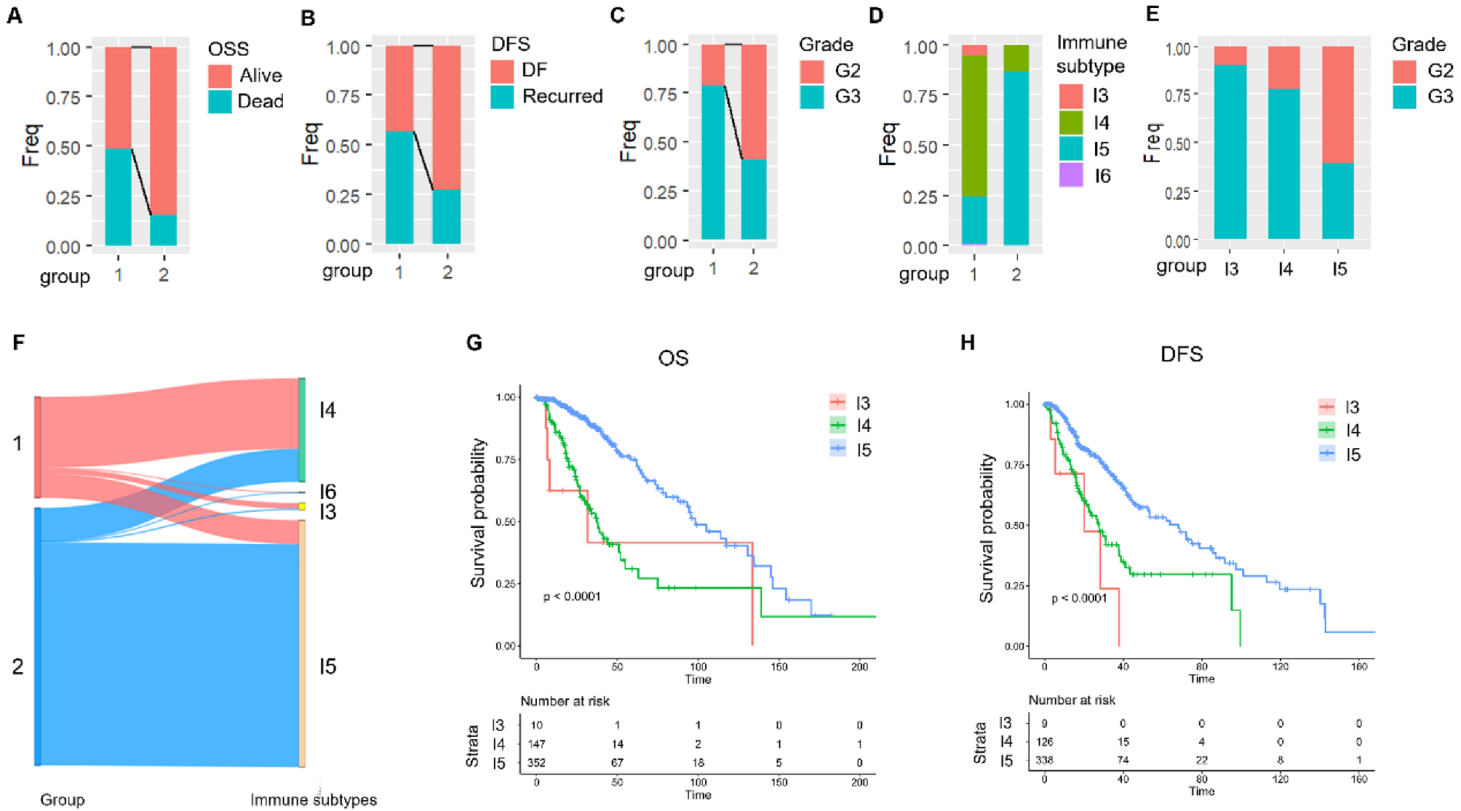
Comparative analysis of clinical features, immune subtypes, and survival outcomes in two molecular subtypes of grade II/III gliomas from TCGA dataset. (**A**–**C**) Compares the distribution of two molecular subtypes in different clinical features in the TCGA dataset. (**D**) Comparison of the distribution of molecular immune subtypes in the two subtypes. (**E**) Compares the distribution of immune subtypes in different clinical features in the TCGA dataset. (**F**) Comparison of the two subtypes with existing molecular immune subtypes. (**G**,**H**) KM curves of OS time and DFS time among different immune subtypes.

We next Compared the different clinical characteristics of the two subtypes and found that: (1) the survival times of these two subtypes were different, and the prognosis in C1 group was worse (Fig. 2A); (2) there was a significant difference in the rate of tumor recurrence between the two subtypes, with a higher proportion of tumor recurrence in group C1 (Fig. 2B). (3) There was a significant difference in the grade proportion between the two subtypes, and the proportion of poor prognosis G3 was higher in group C1 (Fig. 2C).

The six types of immune infiltration identified in human tumors are as follows: Immune cluster 1 (wound healing, I1), Immune cluster 2 (INF-γ dominant, I2), Immune cluster 3 (inflammatory, I3), Immune cluster 4 (lymphocyte depleted, I4), Immune cluster 5 (immunologically quiet, I5), and Immune cluster 6 (TGF-β dominant, I6)^18^. According to the KM curve, I3 and I4 had a worse prognosis than I5 (Fig. 2G,H). Comparing this stratified method with the stratified method of our study (Fig. 2D–F), it was found that I3 and I4 were more distributed in EMT molecular subtype C1, indicating that EMT molecular subtype C1 have more immune subtypes with poor prognosis.

### Poor prognosis subtype may be associated with a higher immune score

To determine the immune-scoring relationship between the two molecular subtypes (C1 and C2), the TCGA data set was analyzed using three immune-scoring software, StromalScore (1.0.13), ImmuneScore (1.0.13), and ESTIMATEScore (1.0.13). MCPcounter can analyze 10 kinds of immune cell scores, while CIBERSOTR can analyze 22 kinds of immune cell scores.

**Figure 3:**
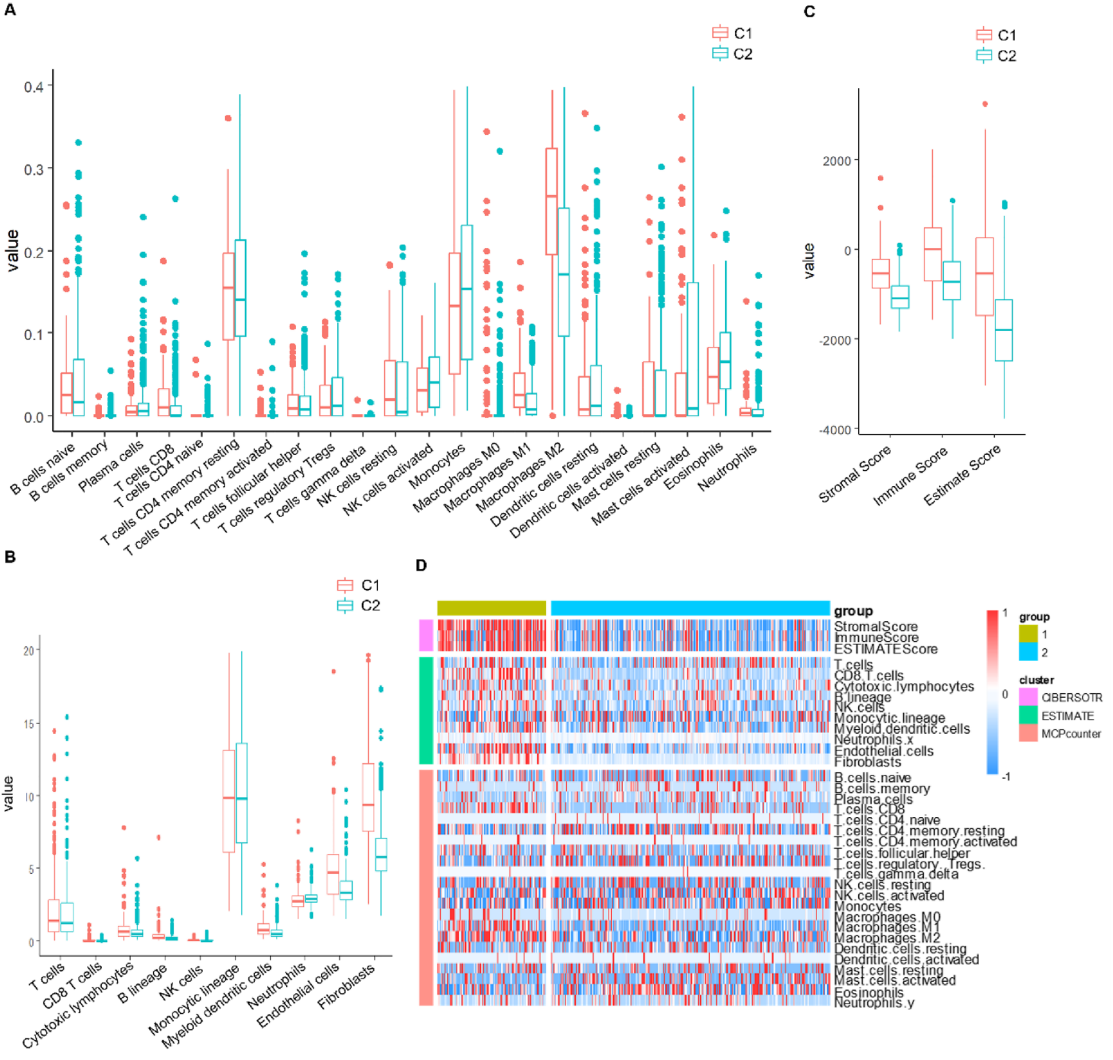
Comparative analysis of immune scores in molecular subtypes of grade II/III gliomas using multiple immune software in TCGA dataset. Comparison of three immune scores among molecular subtypes in TCGA data set. (**A**) Comparison of CIBERSOTR immune scores among molecular subtypes in TCGA data set. (**B**) Comparison of MCPcounter immune scores among molecular subtypes in TCGA dataset. (**C**) ESTIMATE immune scores comparison among molecular subtypes in TCGA dataset. (**D**) Heat map of immune scores comparison among molecular subtypes in TCGA dataset by three immune software.

By comparing the differences in immune scores of the molecular subtypes (C1 and C2) (Fig 3A–C), we found that the immune scores in the C1 subtype were higher than those of the C2 subtype in all three R packages. The heat map of immune scores also showed significantly different immune scores between these two subtypes (Fig. 3D).

### Identification of differentially expressed genes between subtypes

**Figure 4:**
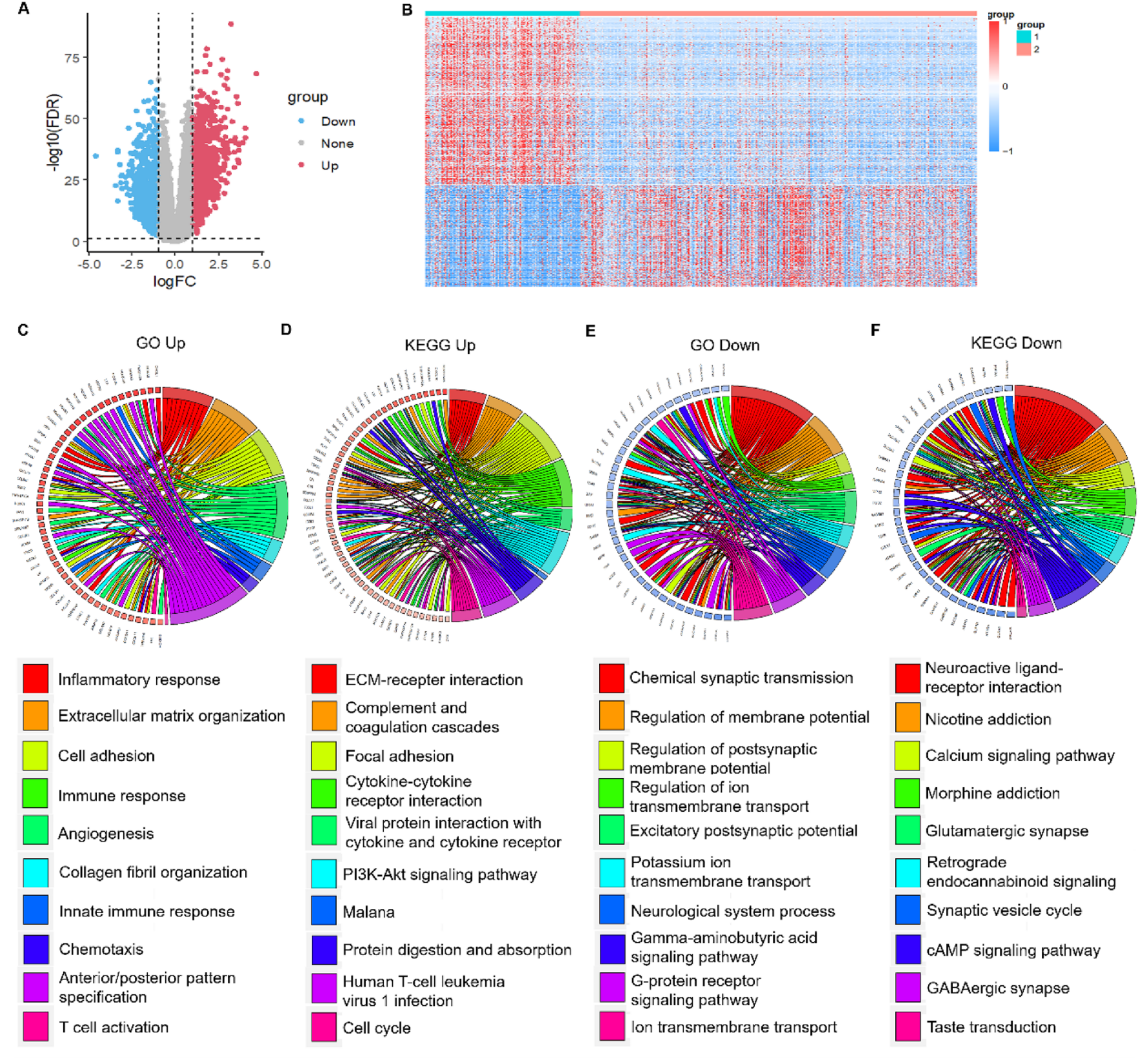
Differential gene expression analysis and functional annotation in molecular subtypes of grade II/III gliomas. (**A**) Volcano map of differentially expressed genes in C1 and C2 groups (**B**) Heat map of differentially expressed genes in 2 groups. (**C**) Biological process (BP) annotation map of differentially up-regulated genes in molecular subtype. (**D**) KEGG annotation map (cite from www.kegg.jp/kegg/kegg1.html) of differentially up-regulated genes in molecular subtypes. (**E**) BP annotation map of differentially down-regulated genes in molecular subtypes. (**F**) KEGG annotation of differentially down-regulated genes in molecular subtypes.

Limma package was then used to calculate molecular subtypes of C1 and C2 DEGs, according to the threshold of | log2FC |> 1 and FDR < 0.01; we screened 1535 DEGs, which contained 1291 up-regulated genes and 244 down-regulated genes. DEGs are shown in Table S2. Genes differentially expressed between C1 and C2 were mainly up-regulated in C1 (Fig. 4A). We selected all the differentially expressed genes and drew a heat map (Fig. 4B).

The Goplot R package was used to perform GO functional enrichment analysis of 1291 up-regulated differentially expressed genes of grade II/III gliomas C1 subtypes. The main pathways enriched were inflammatory response, innate immune response, T cell activation, cell adhesion, and angiogenesis (Fig. 4C).

KEGG pathway enrichment was also performed for the up-regulated differential genes of the C1 subtype. The significantly enriched pathways were extracellular matrix (ECM)-receptor interaction, PI3K-Atk signaling pathway, and cytokine-cytokine receptor interaction (Fig. 4D). Pathway analysis and functional enrichment analysis of 244 down-regulated differential genes were performed using the same method. Significant enrichment of down-regulated genes was observed in the G-protein coupled receptor signaling pathway, neurological system process, Neuroactive ligand-receptor interaction, and Glutamatergic synapse (Fig. 4E,F).

### Construction of predictive risk models

**Figure 5:**
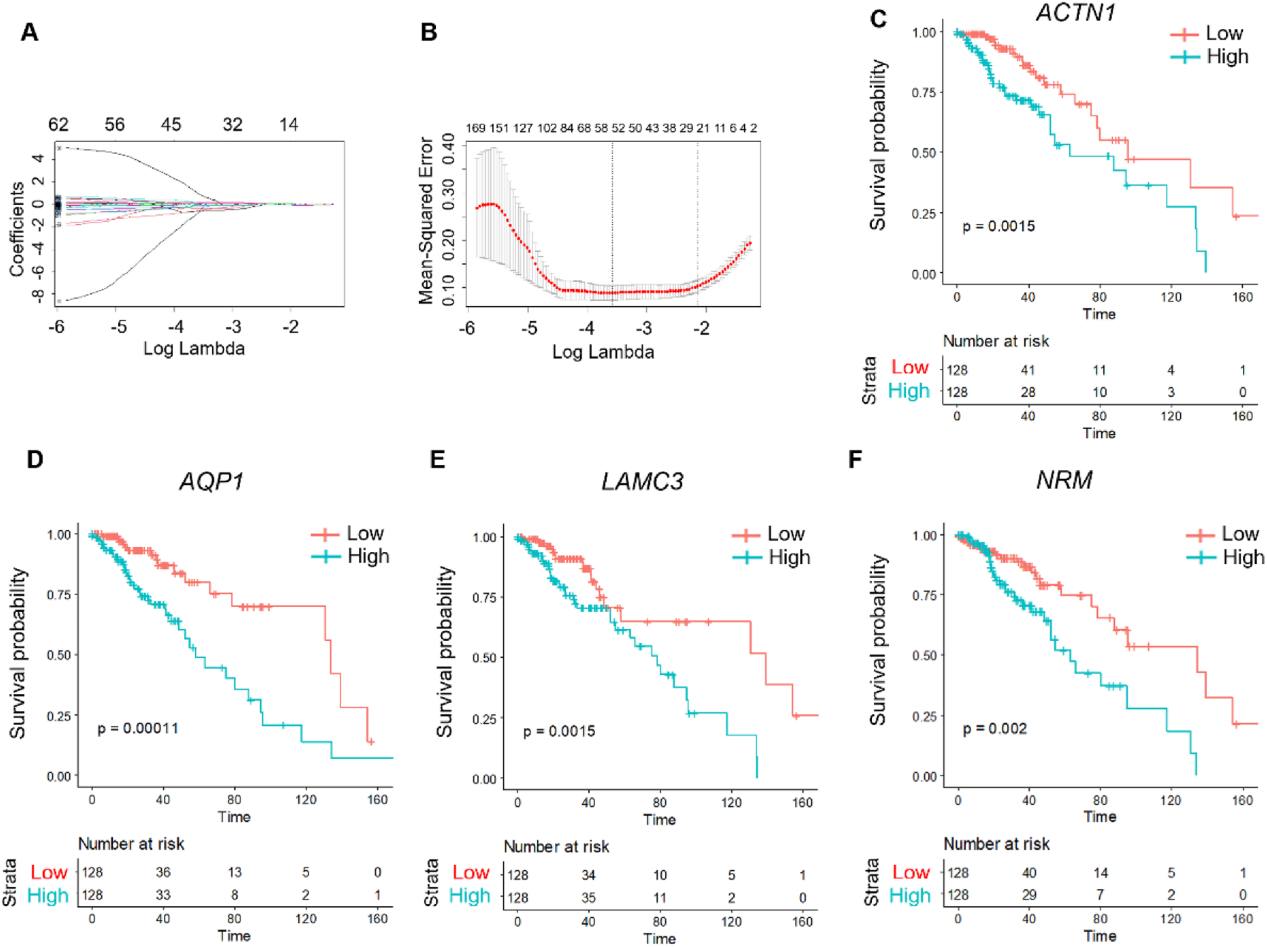
Analysis of independent variables, confidence intervals, and gene survival in TCGA training set. (**A**) Trajectories of independent variables: horizontal axis (log value of a dependent variable) and vertical axis (coefficient of the independent variable). (**B**) Confidence intervals under each λ. (**C**–**F**) KM curves of four genes in TCGA training set.

To further construct the RiskScore model, 512 patient data were randomly grouped. There were 256 samples in both the training set and the test set. First, we calculated the DEGs of C1 and C2, totaling 1864 DEGs. Univariate regression Cox hazard model analysis of survival data revealed that 874 genes were associated with prognosis (*P* < 0.01 was selected as threshold filtering) (Table S3). However, the number of genes was too much for clinical detection. We then used lasso regression to reduce the number of genes further. The R package glmnet was performed for lasso cox regression analysis to analyze the trajectories of independent variables (Fig. 5A). The results showed that the number of coefficients of independent variables gradually increased as lambda increased. Cross-validation was performed to analyze the confidence intervals under each lambda. As shown in Fig. 5B, the model was applicable when lambda = 0.03208551. At this time, when lambda = 0.03208551, 48 genes were selected as the following target genes.

Stepwise regression with the Akaike information criterion (AIC) was then used to account for a statistical fit of the model and parameters. In the MASS package, the AIC method starts with a complex model and removes a variable to reduce it, resulting in a better model with fewer parameters. We used this algorithm and reduced the above 48 genes to 4: ACTN1, AQP1, LAMC3, and NRM. The prognostic KM curves of these four genes were then drawn. As shown in Fig. 5C–F, these four genes were negatively correlated with the survival of samples in the TCGA training set (*P* < 0.05).

**Figure 6:**
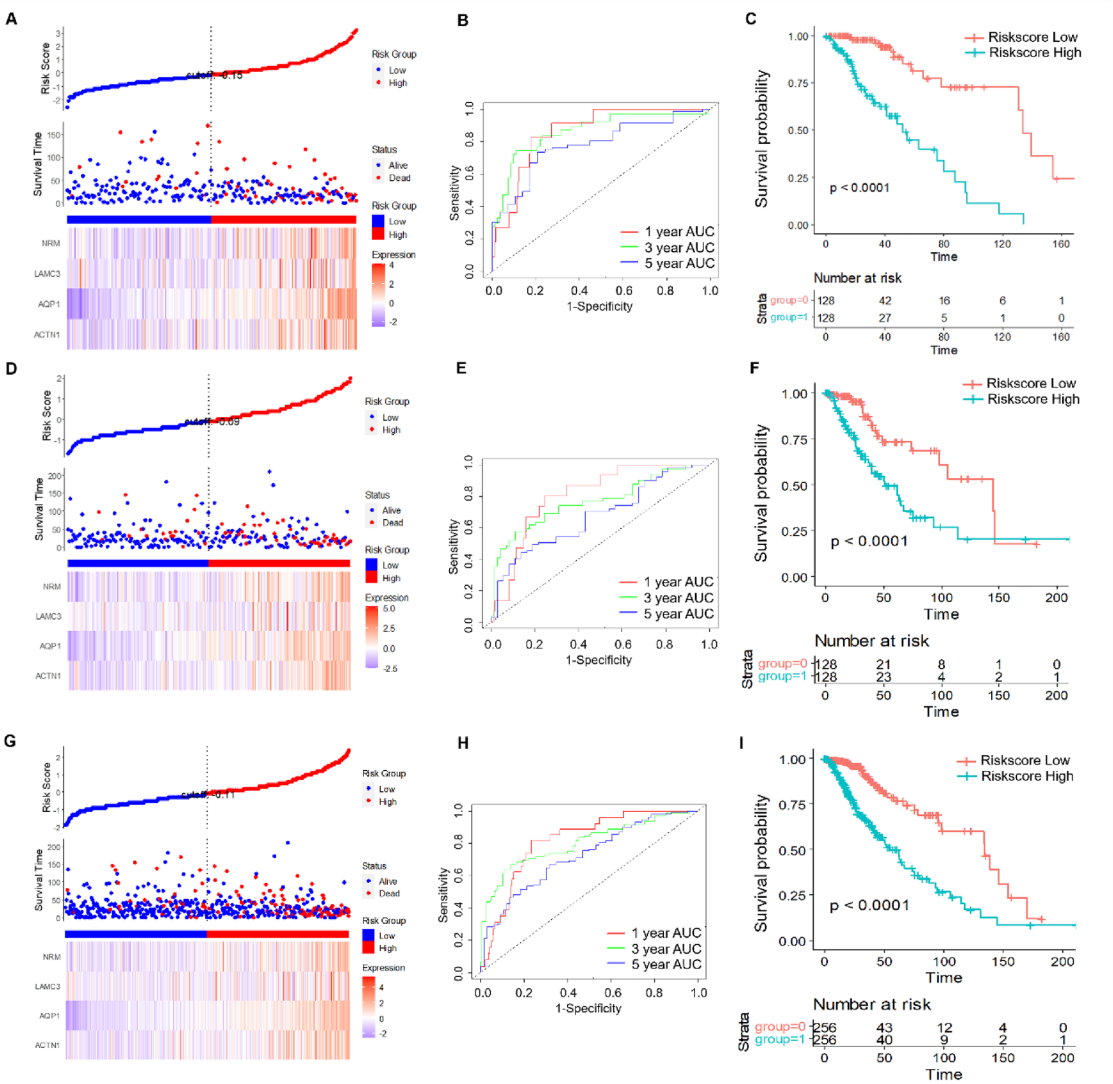
Risk score, gene expression, ROC analysis, and survival distribution in TCGA training, test, and full datasets. Risk score, survival status, and 4 gene expression in the TCGA training set (**A**), the TCGA test set (**D**), and the full TCGA dataset (**G**). ROC curves and AUCs for the 4 gene features in the TCGA training set (**B**), the TCGA test set (**E**), and the full TCGA dataset (**H**). Distribution of KM survival curves for the 4 gene features in the TCGA training set (**C**), the TCGA test set (**F**), and the full TCGA dataset (**I**).

Using the ggRISK package, we calculated a RiskScore for each sample based on the four gene expression levels, and a higher RiskScore indicates a worse prognosis (Fig. 6). The R-wrapper time ROC analysis of the prognostic stratification of RiskScore at 1, 3, and 5 years showed that the model's Area Under Curve (AUC) was higher than 0.75 (Fig. 6B). Finally, the samples were divided into the high RiskScore group and low RiskScore group, and the KM curve was drawn (Fig. 6C). The prognosis of the high RiskScore group was dramatically worse than that of the low RiskScore group, indicating the clinical valve of our RiskScore.

### Validation of risk models

To ascertain the stability of the model, we then conducted the same models and coefficients to analyze the TCGA test dataset. The RiskScore distribution of the TCGA test set (Fig. 6D) suggested that with higher RiskScore, the prognosis of grade II/III glioma patients was worse. In addition, the prognostic prediction efficiency at 1, 3, and 5 years was analyzed using the R package timeROC (Fig. 6E), which showed that the AUC of the model was higher than 0.65. The KM curve showed that the prognosis of the high RiskScore group was dramatically worse than that of the low RiskScore group in the test set (Fig. 6F, P < 0.01).

The RiskScore distribution of the complete TCGA dataset indicated (Fig. 6G) that the prognosis of grade II/III gliomas patients was worse along with higher RiskScore. The prognostic prediction value of RiskScore at 1, 3, and 5 years was validated by the R package timeROC (Fig. 6H), which showed that the AUC of the model was higher than 0.7. The KM curve showed that the prognosis of the high RiskScore group was dramatically worse than that of the low RiskScore group (Fig. 6I, P  < 0.001).

### Verifying the robustness of 4-gene signature external datasets

In order to further clarify the universality of the four gene features we screened, we selected 3 CGGA datasets to validate RiskScore. We used the same models and coefficients in the training set. The RiskScore was calculated separately for each sample based on its mRNA level, and then the RiskScore distribution was plotted.

Consistent with our previous findings, the sample with high RiskScore exhibited a worse prognosis in all three data sets (Supplementary Fig. S1A,D,G). The prognostic prediction efficiency of RiskScore at 1, 3, and 5 years was analyzed using the R package timeROC (Supplementary Fig. S1B,E,H), and the AUC was all higher than 0.7. The KM curve also showed that the prognosis of the high RiskScore group was dramatically worse than that of the low RiskScore group (Supplementary Fig. S1C,F,I).

### Risk model correlation analysis with clinical characteristics and pathways

Four gene features showed that in different age groups, grade groups, IDH mutation stage groups, and gender stage groups, the prognosis of the group with high RiskScore was much worse than that of the group with low RiskScore (Supplementary Fig. S2A–H, *P* < 0.05), suggesting that the model had a strong predictive ability. Comparing the RiskScore distribution among the clinical groups, the grade stage was statistically significant (Supplementary Fig. S2I–J, *P* < 0.05), and a higher RiskScore was associated with a higher grade.

**Figure 7:**
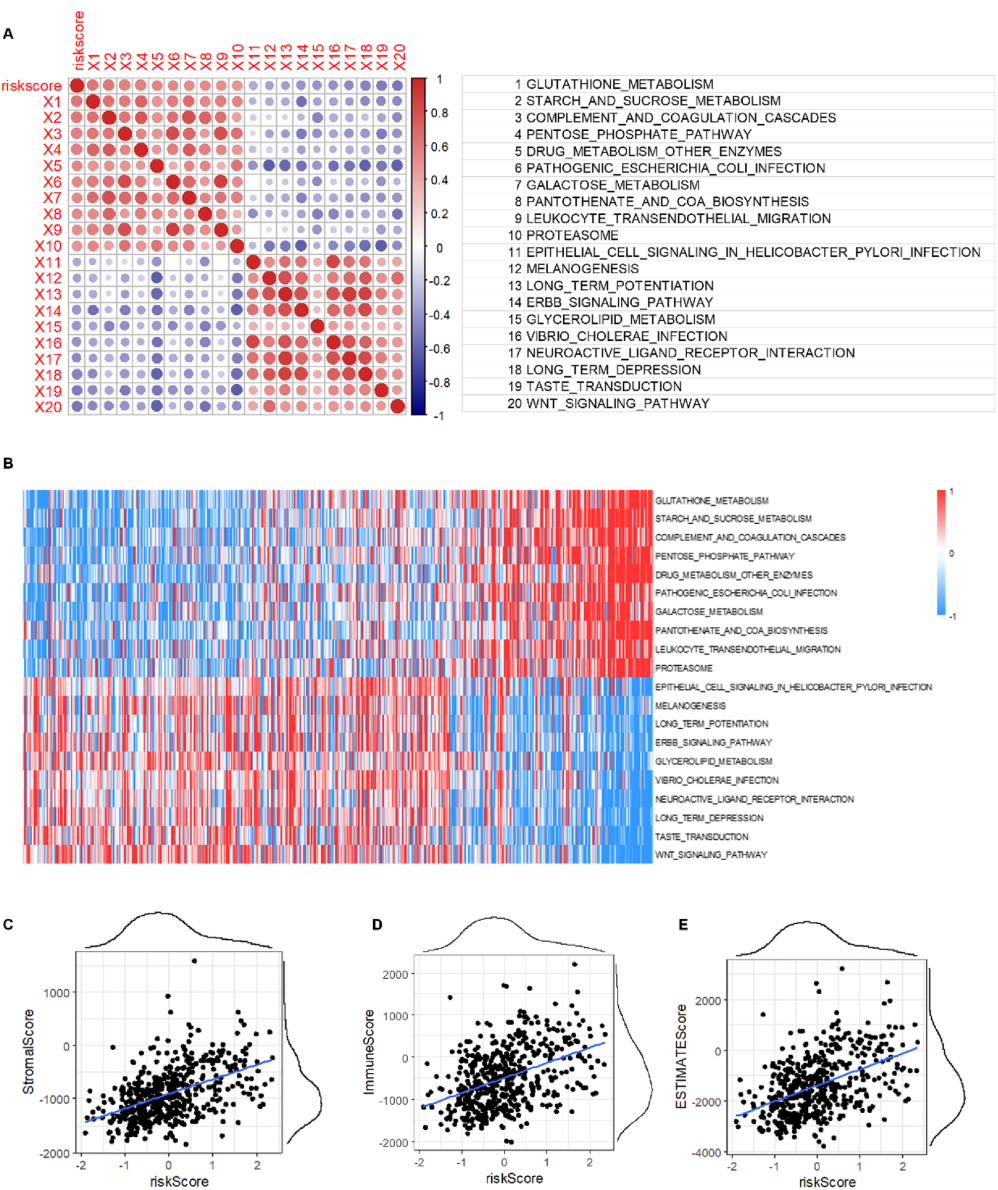
Correlation between ssGSEA and RiskScore. (**A**) Correlation between SSGSEA KEGG signaling pathway score and RiskScore. (**B**) Heat map of the SSGSEA KEGG signaling pathway. (**C**) Correlation between StromalScore and RiskScore. (**D**) Correlation between ImmuneScore and RiskScore. (**E**) Correlation between ESTIMATEScore and RiskScore.

Next, we studied the corresponding gene expression profiles of GSEA to investigate the relationship between the biological function and RiskScores s. First, we calculated the ssGSEA score of each sample in the KEGG pathway. The correlation between the KEGG pathway and RiskScore was then further investigated. Figure 7A,B show 10 KEGG pathways positively correlated with RiskScore, including GLUTATHIONE_METABOLISM, DRUG_METABOLISM_OTHER_ENZYMES, and PATHOGENIC_ESCHERICHIA_COLI_INFECTION, and 10 KEGG pathways that are negatively correlated with RiskScore, including WNT_SIGNALING_PATHWAY, VIBRIO_CHOLERAE_INFECTION, and EPITHELIAL_CELL_SIGNALING_IN_HELICOBACTER_PYLORI_INFECTION.

The R software package analyzed the correlation coefficient between RiskScore and immune score. As shown in Fig. 7C–E, RiskScore was positively correlated with StromalScore, ImmuneScore, and ESTIMATEScore (*P* < 0.05).

### Multivariate COX regression analysis of four-gene signature

**Figure 8:**
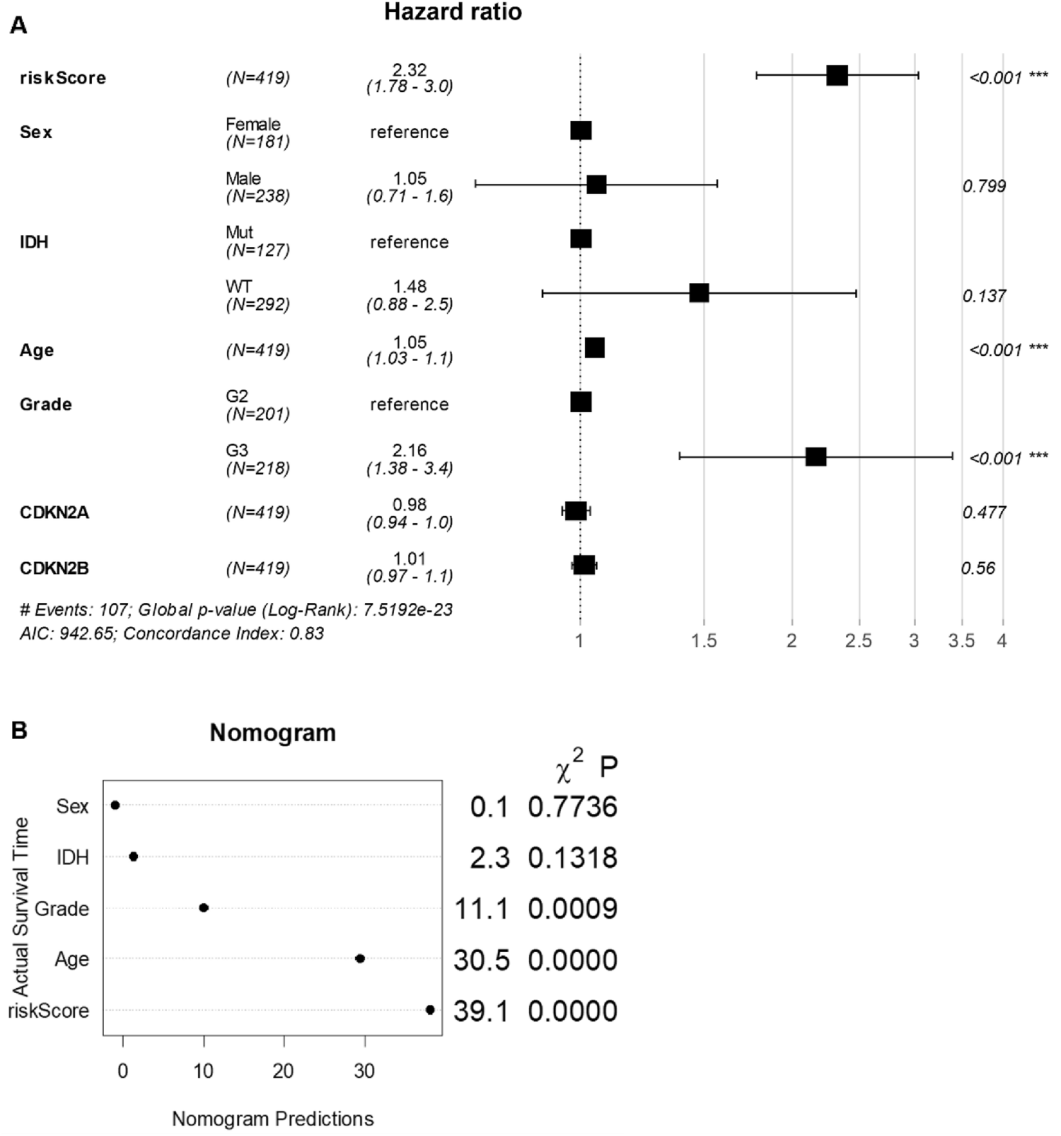
Forest plot and nomogram for Cox regression analysis and prediction in the study population. (**A**) Forest plot of multiple Cox regression analysis. (**B**) Nomogram prediction map based on Sex, IDH, Grade, Age, and riskScore.

The clinical independence of the four-gene features in the TCGA data set was determined by multivariate COX regression analysis, which showed that RiskScore was significantly associated with survival rate (Fig. 8A). We developed a nomogram model to diagnose grade II/III gliomas using a combination of clinical and genetic factors. The nomogram was constructed based on a four-gene signature and clinical factors, including age, sex, and disease stage. Notably, our results showed that among all variables tested, including age, sex, disease stage, and the four-gene signature, the Riskscore had the best performance. These findings suggest that the Riskscore could be a valuable prognostic marker for this disease and may help to guide personalized treatment decisions (Fig. 8B). In summary, our nomogram model provides a useful tool for predicting disease risk and highlights the potential of combining clinical and genetic factors to improve prognostic accuracy. These data together indicate that our four-gene signature model exhibits a promising clinical prediction performance.

### Functions of ACTN1, AQP1, LAMC3 and NRM in proliferation, migration, and invasion of grade II/III glioma cells

**Figure 9:**
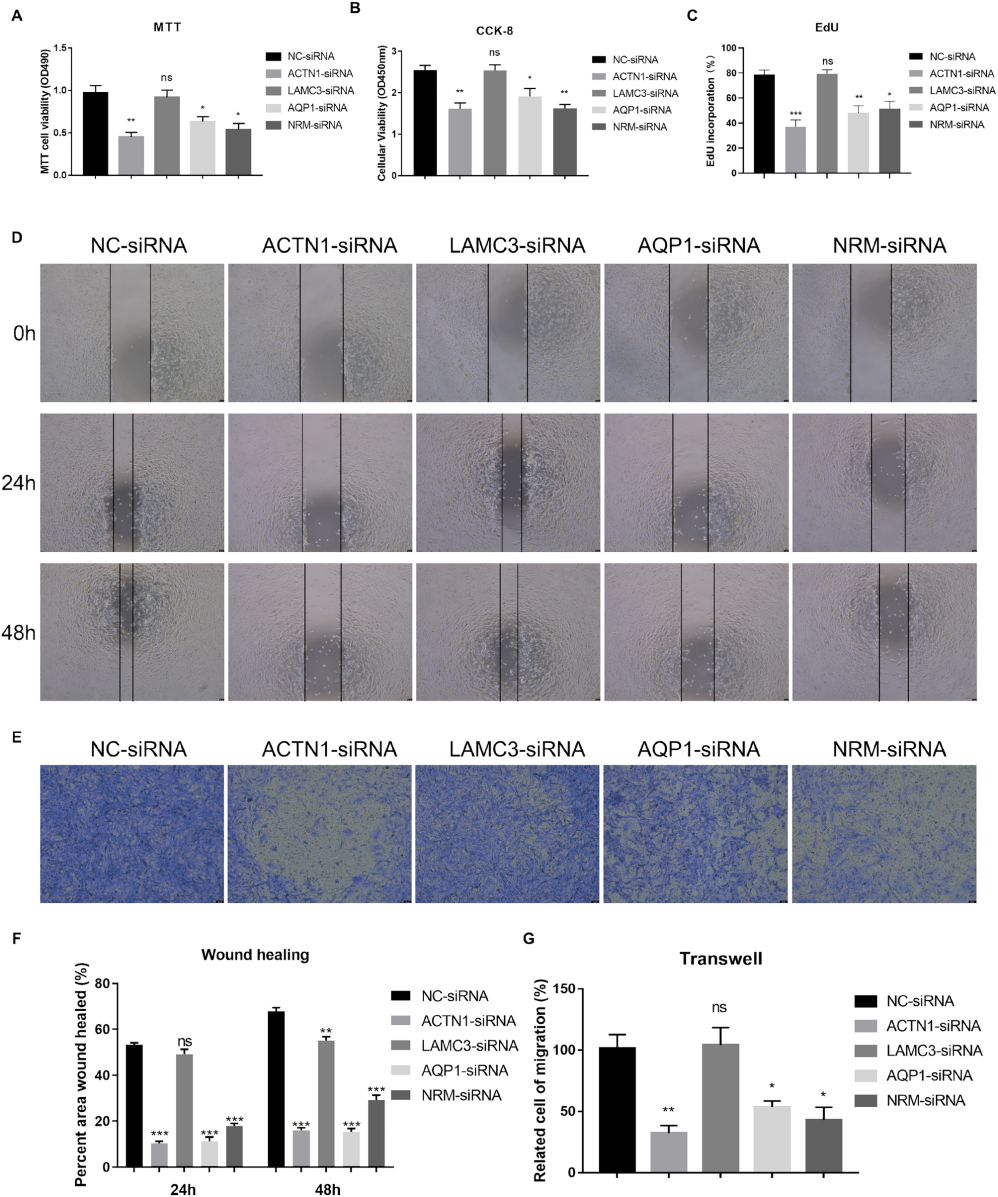
Functions of ACTN1, LAMC3, AQP1, and NRM in proliferation, migration, and invasion of grade II/III glioma cells. (**A**) Effects of knockdown of indicated genes on cell proliferation by MTT assay in SW1088 cells for 24 h. (**B**) Effects of knockdown of indicated genes on cell proliferation by CCK-8 assay in SW1088 cells for 24 h. (**C**) Effects of knockdown of indicated genes on cell proliferation by EdU assay in SW1088 cells for 24 h. (**D**) Effects of knockdown of indicated genes on cell migratory abilities by wound healing assays in SW1088 cells. (**E**) Effects of knockdown of indicated genes on cell invasive capacities by transwell assays in SW1088 cells. (**F**) Statistical results of wound healing assays in (**D**). (**G**) Statistical results of transwell assays in (**E**).

To investigate the functional roles of the four selected genes in grade II/III gliomas, we employed siRNA knockdown of ACTN1, LAMC3, AQP1, and NRM to examine their effects on the proliferation, migration, and invasion capabilities of grade II/III gliomas cell lines (SW1088 cells and SW1733 cells). Using MTT, CCK-8 and EdU assays, we observed that knockdown of ACTN1, AQP1, and NRM significantly inhibited cell proliferation in SW1088 cells and SW1733 cells compared to the control group, indicating the important roles of these genes in promoting cell growth. However, knockdown of LAMC3 did not show significant differences in cell proliferation compared to the control group (Fig. 9A–C, Supplementary Fig. S3C). To assess the effects of gene knockdown on cell migration, we performed wound healing assays. The results revealed that knockdown of ACTN1, AQP1, and NRM significantly impaired cell migration compared to the control group. In contrast, knockdown of LAMC3 did not exhibit a noticeable impact on cell migration capabilities (Fig. 9D,F, Supplementary Fig. S3A,B). Cell invasive capacities were evaluated through transwell assays. Knockdown of ACTN1, AQP1, and NRM led to a substantial decrease in cell invasiveness compared to the control group, highlighting their crucial roles in promoting cell invasion. Conversely, knockdown of LAMC3 did not show a significant difference in cell invasive capacities compared to the control group (Fig. 9E,G). In summary, our findings demonstrate that knockdown of ACTN1, AQP1, and NRM suppresses cell proliferation, migration, and invasion in SW1088 cells. In contrast, knockdown of LAMC3 does not exhibit significant alterations in these cellular processes. These results emphasize the important roles of ACTN1, AQP1, and NRM in regulating the proliferation, migration, and invasion of SW1088 cells, providing valuable insights into their potential as therapeutic targets for inhibiting tumor progression and metastasis.

### ACTN1 expression correlates with T cell exhaustion and negatively affects prognosis in grade II/III glioma patients

**Figure 10:**
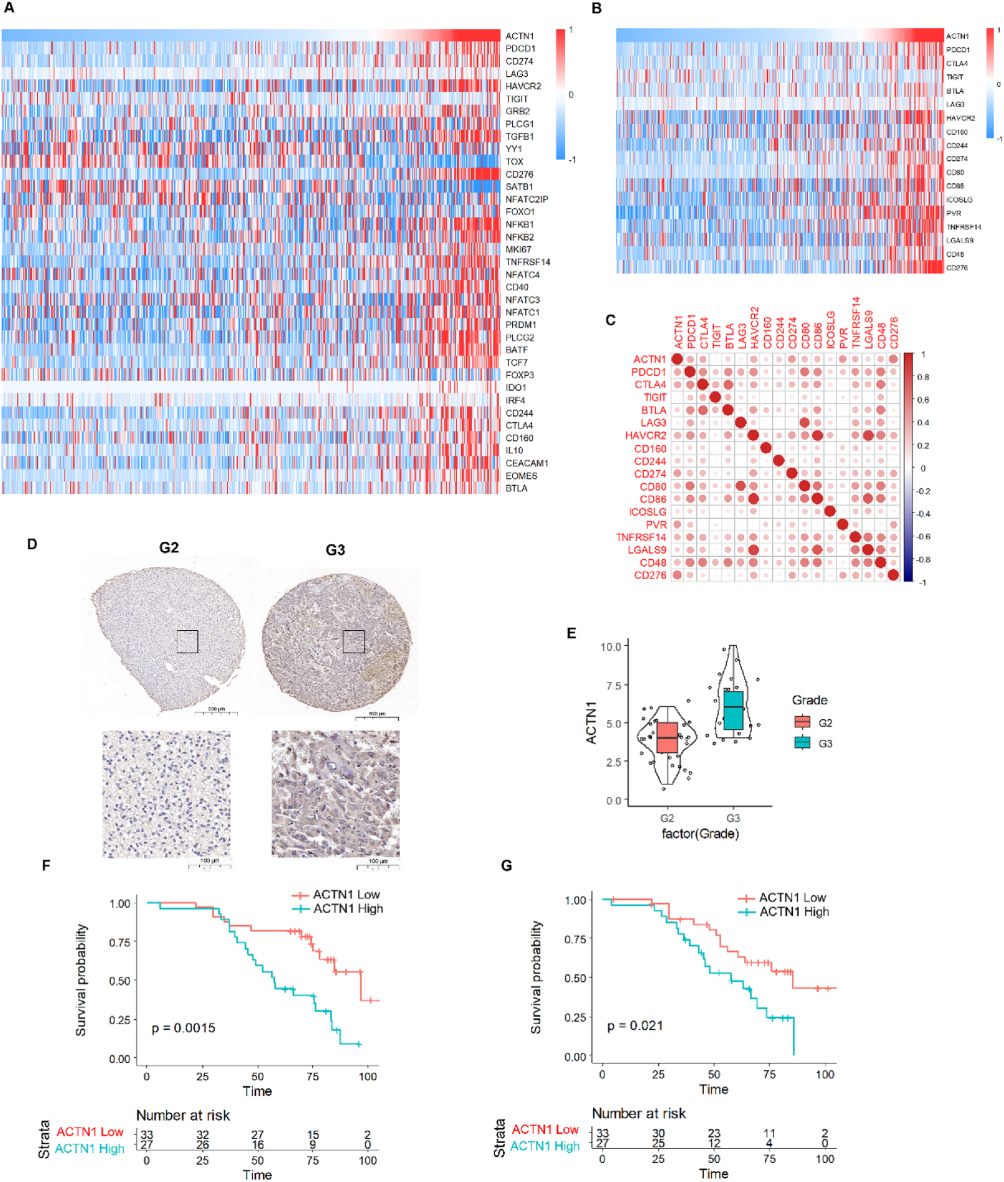
Positive correlation between ACTN1 expression and exhaustion-related molecules. (**A**) Heatmap of the correlation between ACTN1 expression and the expression of 39 exhaustion-related molecules, TCGA samples sorted by ACTN1 expression. (**B**) Heatmap of the correlation between ACTN1 expression and the expression of immune checkpoints and their receptors, TCGA samples sorted by ACTN1 expression. (**C**) Correlation between ACTN1 expression and immune checkpoints and their receptors. (**D**,**E**) Protein expression of ACTN1 in glioma tissue samples. (**F**) Kaplan–Meier curve of overall survival (OS) according to ACTN1 protein expression levels. (**G**) Kaplan–Meier curve of disease-specific survival (DSS) according to ACTN1 protein expression levels.

ACTN1 is highly expressed in various tumors and has a known impact on the prognosis of cancer patients. In the context of the four-gene signature's relationship with immunity and its correlation with grade II/III gliomas patient prognosis, we explored the potential link between ACTN1 and T cell exhaustion within tumors. Given that we have discovered a close association between the four-gene signature and immunity and that ACTN1 expression is negatively correlated with the prognosis of grade II/III glioma patients, we hypothesize that ACTN1 may affect T-cell exhaustion within tumors. We examined correlations of the four-gene signature genes with T cell exhaustion markers and found ACTN1 expression had the strongest association (Supplementary Fig. S4A–E). Through literature review of 39 genes related to T-cell exhaustion, we found that ACTN1 is positively correlated with the expression of most genes (Fig. 10A). Immune checkpoints refer to molecules that can regulate immune responses. They have specific functions in normal cells but can be exploited in tumors. Immune checkpoint molecules can modulate immune responses by interacting with other proteins. The most common immune checkpoint molecules include PD-1, TIM3, and LAG-3. Furthermore, we conducted a correlation analysis between the expression of recently discovered immune checkpoint molecules and their ligands and ACTN1 expression. We found that ACTN1 expression is positively correlated with immune checkpoint molecules and their ligands (Fig. 10B,C). In summary, we have found that ACTN1 is closely related to grade II/III gliomas prognosis, and its expression is positively correlated with T cell exhaustion-related gene expression, indicating that ACTN1 may affect T cell exhaustion within tumors. Our previous data have demonstrated a negative correlation between ACTN1 mRNA expression and grade II/III gliomas prognosis. Based on this, we further validated the clinical relevance of ACTN1 protein expression using immunohistochemistry and found that ACTN1 expression is negatively correlated with grade II/III gliomas patient prognosis (Fig. 10D–G).

In conclusion, ACTN1 is highly expressed in various tumors, and its expression is negatively correlated with grade II/III gliomas patient prognosis. ACTN1 may affect T cell exhaustion within tumors, and its expression is positively correlated with the expression of T cell exhaustion-related genes. Furthermore, we have found that ACTN1 expression is positively correlated with immune checkpoint molecules and their ligands. Immunohistochemistry further validates the clinical relevance of ACTN1 protein expression, suggesting that ACTN1 may serve as an important prognostic marker in grade II/III gliomas. These findings may contribute to a deeper understanding of the mechanism underlying grade II/III gliomas development, improve the diagnosis and treatment of grade II/III gliomas, and guide the development of immune therapies.

## Discussion

Patients with grade II/III gliomas usually lack apparent clinical symptoms in the early disease stage. The treatment of these patients remains a significant public health challenge worldwide due to the high morbidity and mortality of the disease. Given this significant heterogeneity in the natural history and prognosis of grade II/III gliomas, identification of robust molecular markers that can accurately stratify patients and predict clinical outcomes is imperative to guide clinical decision-making and improve patient survival. Although previous studies have proposed prognostic models for gliomas, prognostication specifically for the grade II/III gliomas subgroup based on underlying biological processes such as EMT has been lacking. Our study aimed to leverage EMT-related gene expression patterns to uncover molecular grade II/III glioma subtypes with distinct prognostic outcomes. Furthermore, we have identified and validated a novel four-gene signature that serves as an independent prognostic marker for grade II/III gliomas. The clinical implementation of such robust prognostic markers can assist in patient counseling, treatment planning, and enrollment in clinical trials to ultimately improve grade II/III gliomas patient outcomes.

In our study, 512 grade II/III glioma samples were classified into two molecular subtypes with different clinical features and prognostic outcomes based on 132 prognostic EMT-related genes identified from TCGA. Overall, group C1 had a worse prognosis, a higher proportion of deaths, a higher grade, and a higher immune score than group C2. On this basis, a prognostic evaluation model was constructed to distinguish different molecular subtypes and evaluate the prognosis of grade II/III glioma patients.

Recently, many studies have highlighted several prognostic tumor models. However, few studies have focused on screening grade II/III gliomas prognostic markers based on EMT-related genes. In this study, we constructed a novel and highly robust four-gene signature (ACTN1, AQP1, LAMC3, and NRM) based on EMT-related genes to predict grade II/III gliomas prognosis. We validated this marker in three additional independent databases.

ACTN1 has been reported to predict poor prognosis of acute lymphoblastic leukemia, oral squamous cell carcinoma, and breast cancer^19^. Oroxylin A, designed for targeting ACTN1, can remodel the interstitial microenvironment of breast cancer and inhibit tumor metastasis^20^. Furthermore, the protein level of ACTN1 has been reported closely related to tumor growth, TNM stage, and prognosis in hepatocellular carcinoma (HCC)^21^. In vitro and in vivo experiments have shown that ACTN1 is essential for HCC growth. These results suggest that ACTN1 plays an essential role in the development and progression of HCC^21^. Knockdown of ACTN1 could increase the phosphorylation levels of YAP and LATS1, thereby inhibiting their loss of transcriptional activation in the cytoplasm.

Furthermore, these results were confirmed by examining alterations in classical YAP target genes. YAP/TAZ has become an attractive target for cancer treatment. Therefore, targeting ACTN1-mediated YAP activation may serve as a potential approach to cancer therapy. Indeed, blocking the YAP-TEAD axis can significantly inhibit ACTN1-mediated tumor growth^21^. Moreover, α-Actin is essential for forming microfilaments in contractile filaments or stress fibers, and ACTN1 deletion contributed to inhibiting the actin regulators RhoA and CDC42, two actin regulators. In addition, the knockdown of ACTN1 affects the F-actin organization in HCC cells^22^. Therefore, ACTN1 may promote HCC cell migration through a Hippo-independent molecular mechanism.

We validated the clinical relevance of ACTN1 protein expression using immunohistochemistry and found that ACTN1 expression is negatively correlated with grade II/III gliomas patient prognosis. We further found that ACTN1 is closely related to grade II/III gliomas prognosis, and its expression is positively correlated with T cell exhaustion-related gene expression, indicating that ACTN1 may affect T cell exhaustion within tumors. Based on this, we believe that ACTN1 could be used as a target for regulating immune checkpoints, such as PD-1, TIM3, and LAG-3, to treat grade II/III gliomas.

AQP1 is a member of water transport selective channels, and its function in osmotic gradients and maintaining tissue water balance has been well-established. Recently it has been considered an oncogene linked with various tumors, including breast cancer^23,24^. Tumor cells expressing a high level of AQP1 exhibit higher proliferation and migration rates, resulting in more extensive tumors and more frequent lung metastases in vivo. This suggests that AQP1 is critical in acquiring an aggressive phenotype in TNBC^25^. The classical paradigm for AQP-induced cell growth and invasion has been proposed as AQP polarization, which promotes cell morphological changes and directed migration by coordinating AQP-dependent transmembrane water/ion flow in the osmotic gradient. This "osmotic engine model" perfectly explains how membrane AQP1 regulates tumor migration and angiogenesis. Nevertheless, the role of AQP1 enriched in the cytoplasm remains elusive, which might be essential to decipher the mechanisms of tumor progression and metastasis^25^. Emerging evidence has recently identified that AQP-1-driven tumors resist necroptosis and apoptosis, which may contribute to AQP1-mediated tumor proliferation and metastasis^25^.

LAMC3 (laminin subunit Gamma 3) is a protein-coding gene. Disorders associated with LAMC3 include cortical malformations, occipital, and polymicrogyria^26^. The related pathways include the integrin pathway and ERK signaling pathway. Laminins, a family of extracellular matrix glycoproteins, are the major noncollagenous component of basement membranes. Proteins in this family are involved in various biological processes, including differentiation, cell adhesion, neurite outgrowth, and migration. Gamma 3 is also an essential component of the apical surface of ciliated epithelial cells in the lung, vas deferens, fallopian tubes, epididymis, and seminiferous tubules^27^. Low expression of LAMC3 is associated with malignant progression and poor prognosis of ovarian cancer (OC), which is expected to become a new therapeutic target and prognostic marker for clinical treatment^28^. Currently, the study of LAMC3 in other tumors is relatively few, and it is worthy of further study.

NRM-encoded proteins contain transmembrane domains, reside within the inner nuclear membrane of the nucleus, and are tightly associated with the nucleus. This protein has homology with isoprenyl cysteine carboxyl methyltransferase alternative splicing results in multiple transcriptional variants encoding different protein isoforms^29^. Currently, the study of NRM in tumors is very few, and it is worthy of further study.

In this study, we found that the four-gene signature is dispensable in a wide range of tumorigenic processes, such as tumor growth and metastasis, making the four-gene marker construct a potential biomarker for predicting the prognosis of grade II/III gliomas.

The evaluation of the four-gene structure by GSEA revealed that the tumor features were significantly rich while metabolic profiles varied. GSEA analysis revealed that many tumor-related pathways were enriched overexpressed in subtype C1, suggesting that C1 subtype is more malignancy. This finding is also confirmed by the clinical characteristics that the C1 group has an advanced stage and high mortality. Interestingly, the mRNA levels of metabolism-related genes in the C2 group were higher than those in the C1 group. Most of these metabolic pathways were as follows: glutathione metabolism and drug metabolism. In addition, we found that tumor-related pathways increased with RiskScore. In contrast, metabolic-related pathways decreased with RiskScore, suggesting that RiskScores can help predict the prognosis of grade II/III gliomas and provide a probable explanation for the molecular basis of grade II/III gliomas progression and occurrence.

Despite the encouraging results of our study, there are certain limitations. First, our finding is based on a single platform, and the study is retrospective. Although our model is well validated in the CGGA database, data from different constitutions and platforms are needed to validate our model's performance further. Second, the limited sample size may have led to selection bias. Third, our procedure for screening differentially expressed genes was primarily based on statistics. Therefore, specific genes of biological significance may have been ignored. Finally, the four genes identified in this study (including ACTN1, AQP1, LAMC3, and NRM) need to be separately explored in animal models to validate their roles in EMT further and lay the foundation for clinical application.

### Supplementary Information

Supplementary Figures.

Supplementary Tables.

## Data Availability

The datasets used and/or analyzed during the current study available from the corresponding author on reasonable request.

## References

[1] Anandappa AJ, Wu CJ, Ott PA (2020). Directing traffic: How to effectively drive T cells into tumors. Cancer Discov..

[2] Siegel RL, Miller KD, Wagle NS, Jemal A (2023). Cancer statistics. CA Cancer J. Clin..

[3] Tanaka S, Louis DN, Curry WT, Batchelor TT, Dietrich J (2013). Diagnostic and therapeutic avenues for glioblastoma: No longer a dead end?. Nat. Rev. Clin. Oncol..

[4] Cloughesy TF, Cavenee WK, Mischel PS (2014). Glioblastoma: From molecular pathology to targeted treatment. Annu. Rev. Pathol..

[5] Han J, Khatwani N, Searles TG, Turk MJ, Angeles CV (2020). Memory CD8(+) T cell responses to cancer. Semin. Immunol..

[6] Mair MJ, Geurts M, van den Bent MJ, Berghoff AS (2021). A basic review on systemic treatment options in WHO grade II-III gliomas. Cancer Treat Rev..

[7] Kunimatsu A, Kunimatsu N, Kamiya K, Watadani T, Mori H, Abe O (2018). Comparison between glioblastoma and primary central nervous system lymphoma using MR image-based texture analysis. Magn. Reson. Med. Sci..

[8] Brooks LJ, Clements MP, Burden JJ, Kocher D, Richards L, Devesa SC, Zakka L, Woodberry M, Ellis M, Jaunmuktane Z, Brandner S, Morrison G, Pollard SM, Dirks PB, Marguerat S, Parrinello S (2021). The white matter is a pro-differentiative niche for glioblastoma. Nat. Commun..

[9] Pastushenko I, Blanpain C (2019). EMT transition states during tumor progression and metastasis. Trends Cell Biol..

[10] Brabletz S, Schuhwerk H, Brabletz T, Stemmler MP (2021). Dynamic EMT: A multi-tool for tumor progression. EMBO J..

[11] Chen T, You Y, Jiang H, Wang ZZ (2017). Epithelial-mesenchymal transition (EMT): A biological process in the development, stem cell differentiation, and tumorigenesis. J. Cell Physiol..

[12] Saitoh M (2018). Involvement of partial EMT in cancer progression. J. Biochem..

[13] Jiang J, Chen H-N, Jin P, Zhou L, Peng L, Huang Z, Qin S, Li B, Ming H, Luo M, Xie N, Gao W, Nice EC, Yu Q, Huang C (2023). Targeting PSAT1 to mitigate metastasis in tumors with p53-72Pro variant. Sig. Transduct Target Ther..

[14] Bergman DR, Karikomi MK, Yu M, Nie Q, MacLean AL (2021). Modeling the effects of EMT-immune dynamics on carcinoma disease progression. Commun. Biol..

[15] Mahmoudian RA, Mozhgani S, Abbaszadegan MR, Mokhlessi L, Montazer M, Gholamin M (2021). Correlation between the immune checkpoints and EMT genes proposes potential prognostic and therapeutic targets in ESCC. J. Mol. Histol..

[16] Nesvick CL, Zhang C, Edwards NA, Montgomery BK, Lee M, Yang C, Wang H, Zhu D, Heiss JD, Merrill MJ, Ray-Chaudhury A, Zhuang Z (2016). ZEB1 expression is increased in IDH1-mutant lower-grade gliomas. J. Neurooncol..

[17] Kanehisa M, Furumichi M, Sato Y, Kawashima M, Ishiguro-Watanabe M (2023). KEGG for taxonomy-based analysis of pathways and genomes. Nucleic Acids Res..

[18] Thorsson V, Gibbs DL, Brown SD, Wolf D, Bortone DS, Ouyang TH, Porta-Pardo E, Gao GF (2018). The immune landscape of cancer. Immunity.

[19] Xie GF, Zhao LD, Chen Q, Tang DX, Chen QY, Lu HF, Cai JR, Chen Z (2020). High ACTN1 is associated with poor prognosis, and ACTN1 silencing suppresses cell proliferation and metastasis in oral squamous cell carcinoma. Drug Des. Devel Ther..

[20] Cao Y, Cao W, Qiu Y, Zhou Y, Guo Q, Gao Y, Lu N (2020). Oroxylin A suppresses ACTN1 expression to inactivate cancer-associated fibroblasts and restrain breast cancer metastasis. Pharmacol. Res..

[21] Chen Q, Zhou XW, Zhang AJ, He K (2021). ACTN1 supports tumor growth by inhibiting Hippo signaling in hepatocellular carcinoma. J. Exp. Clin. Cancer Res..

[22] Quick Q, Skalli O (2010). Alpha-actinin 1 and alpha-actinin 4: Contrasting roles in the survival, motility, and RhoA signaling of astrocytoma cells. Exp. Cell Res..

[23] Kao SC, Armstrong N, Condon B, Griggs K, McCaughan B, Maltby S, Wilson A, Henderson DW, Klebe S (2012). Aquaporin 1 is an independent prognostic factor in pleural malignant mesothelioma. Cancer.

[24] Yamazato Y, Shiozaki A, Ichikawa D, Kosuga T, Shoda K, Arita T, Konishi H, Komatsu S, Kubota T, Fujiwara H, Okamoto K, Kishimoto M, Konishi E, Marunaka Y, Otsuji E (2018). Aquaporin 1 suppresses apoptosis and affects prognosis in esophageal squamous cell carcinoma. Oncotarget.

[25] Yin Z, Chen W, Yin J, Sun J, Xie Q, Wu M, Zeng F, Ren H (2021). RIPK1 is a negative mediator in Aquaporin 1-driven triple-negative breast carcinoma progression and metastasis. NPJ Breast Cancer.

[26] Urgen BM, Topac Y, Ustun FS, Demirayak P, Oguz KK, Kansu T, Saygi S, Ozcelik T, Boyaci H, Doerschner K (2019). Homozygous LAMC3 mutation links to structural and functional changes in visual attention networks. Neuroimage.

[27] Zambonin JL, Dyment DA, Xi Y, Lamont RE, Hartley T, Miller E, Kerr M, Boycott KM, Parboosingh JS, Venkateswaran S (2018). A novel mutation in LAMC3 associated with generalized polymicrogyria of the cortex and epilepsy. Neurogenetics.

[28] Lei SM, Liu X, Xia LP, Ke Y, Wei LW, Li L, Yin FJ (2021). Relationships between decreased LAMC3 and poor prognosis in ovarian cancer. Zhonghua Fu Chan Ke Za Zhi.

[29] Hofemeister H, O'Hare P (2005). Analysis of the localization and topology of nurim, a polytopic protein tightly associated with the inner nuclear membrane. J. Biol. Chem..

