# Supplementary material for: Identification of EMT-associated prognostic features among grade II/III gliomas
Source: Sci Rep. 2024 Feb 3;14:2822. doi: 10.1038/s41598-024-53399-0 (PMC10837424; doi:10.1038/s41598-024-53399-0)
Supplement: Supplementary file 1 — Supplementary Figures. [file 41598_2024_53399_MOESM1_ESM.docx]

**Supplementary** **Figure**


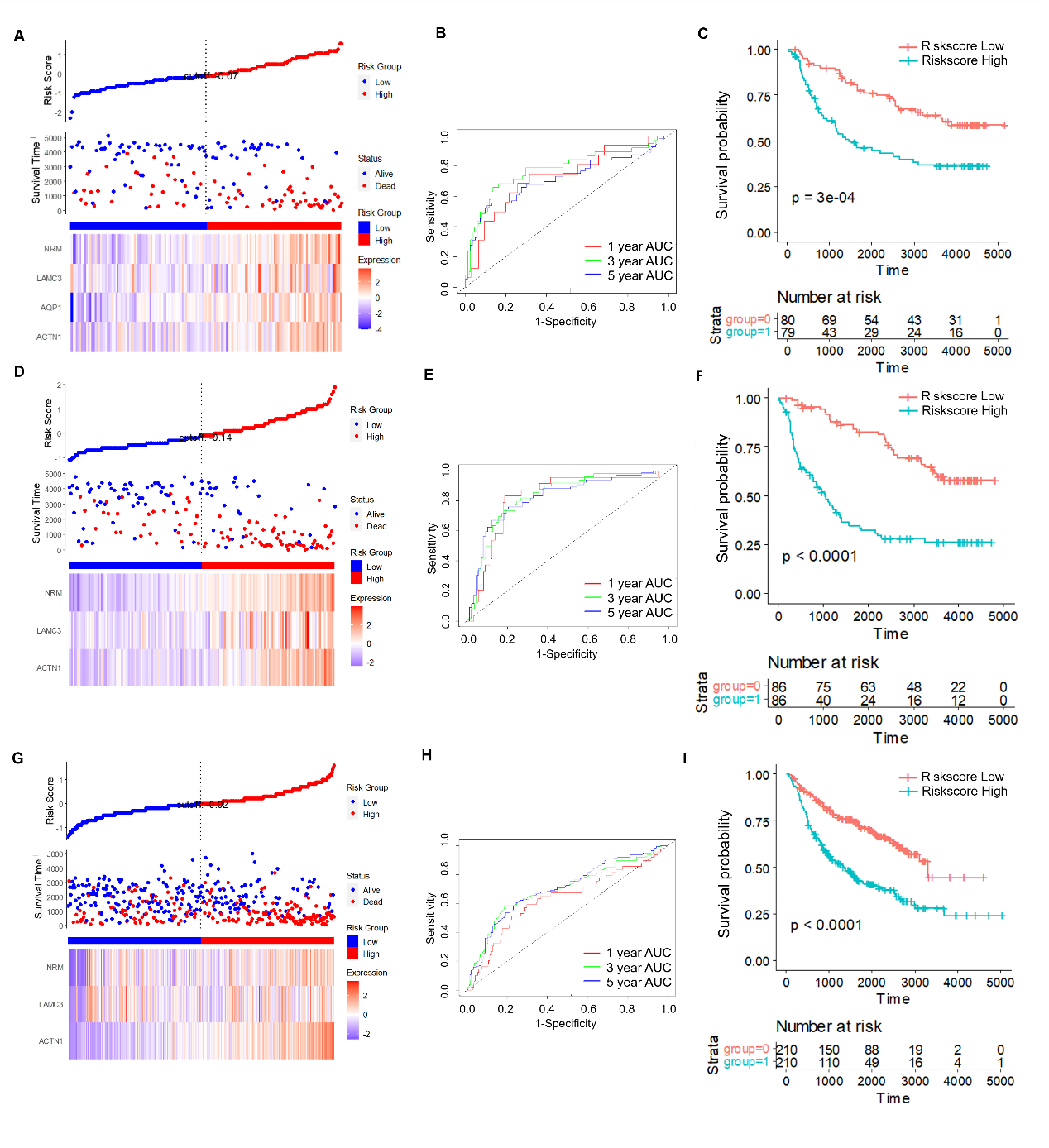


**Supplementary** **Figure 1. Riskscores, gene expression, ROC curves, and KM survival curves in different CGGA databases.** Risk score, survival status, and 4 gene expression in the CGGA-mRNA-array_301 database **(A)**, the CGGA-mRNAseq_325 database **(D)**, and the CGGA-mRNAseq_693 database **(G)**. ROC curves and AUCs for the 4 gene features in the CGGA-mRNA-array_301 database **(B)**, the CGGA-mRNAseq_325 database **(E)**, and the CGGA-mRNAseq_693 database **(H)**. Distribution of KM survival curves for the 4 gene features in the CGGA-mRNA-array_301 database **(C)**, the CGGA-mRNAseq_325 database **(F)**, and the CGGA-mRNAseq_693 database **(I)**.

**
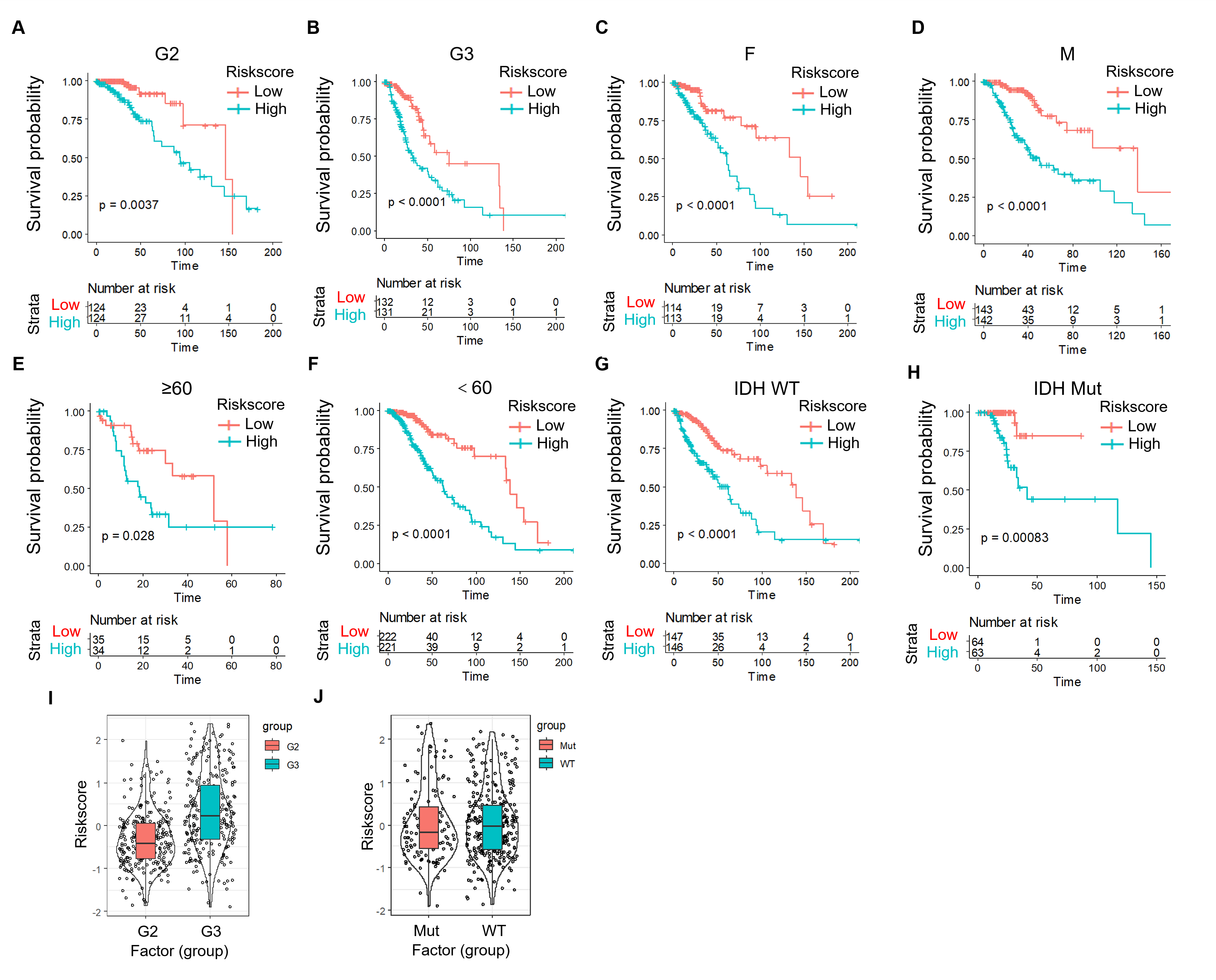
**

**Supplementary** **Figure 2. Prognostic performance analysis of risk models based on clinical characteristics. A.** According to RiskScore, the patients in the G2 group were divided into two groups with significant prognosis. **B.** According to RiskScore, patients in the G3 group were divided into two groups with significant prognosis. **C.** According to RiskScore, the female group was divided into two groups with significant prognosis. **D.** Patients in the male group were divided into two groups according to RiskScore. **E.** Patients aged ≥60 years were divided into two groups with significant prognosis according to RiskScore. **F.** According to RiskScore, patients with ages < 60 were divided into two groups with significant prognosis. **G.** Patients in IDH WT group were divided into two groups according to RiskScore, and the prognosis was significant. **H.** Patients with IDH mutation were divided into two groups according to RiskScore, and the prognosis was significant. **I.** RiskScore scores in G2 and G3. **J.** RiskScore scores in IDH WT and IDH mutation groups.


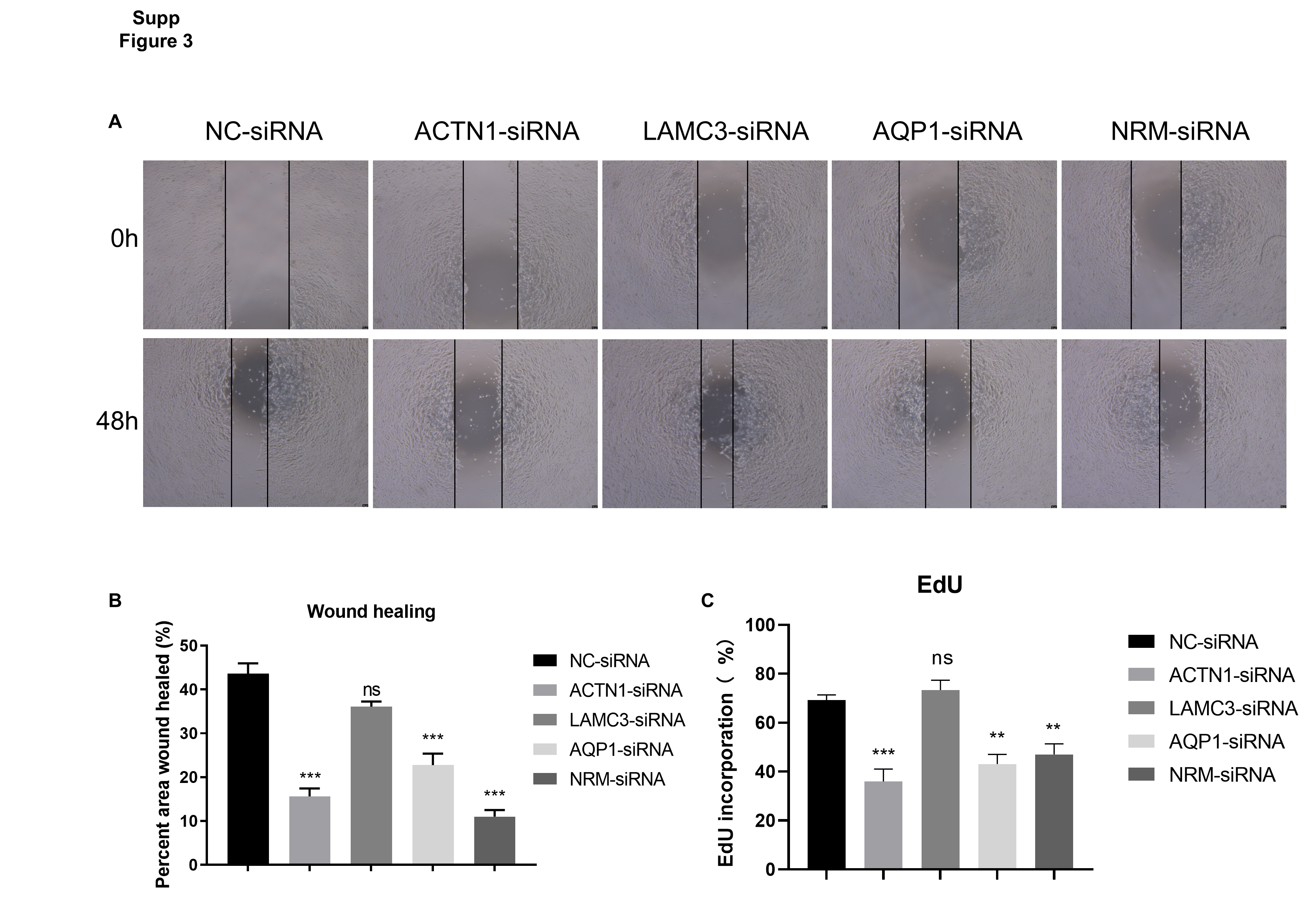


**Supplementary** **Figure 3. Functions of ACTN1, LAMC3, AQP1, and NRM in proliferation and migration of grade II/III glioma cells.** **A.** Effects of knockdown of indicated genes on cell migratory abilities by wound healing assays in SW1733 cells. **B.** Statistical results of wound healing assays in **(A). C.** Effects of knockdown of indicated genes on cell proliferation by EdU assay in SW1733 cells for 24 h.


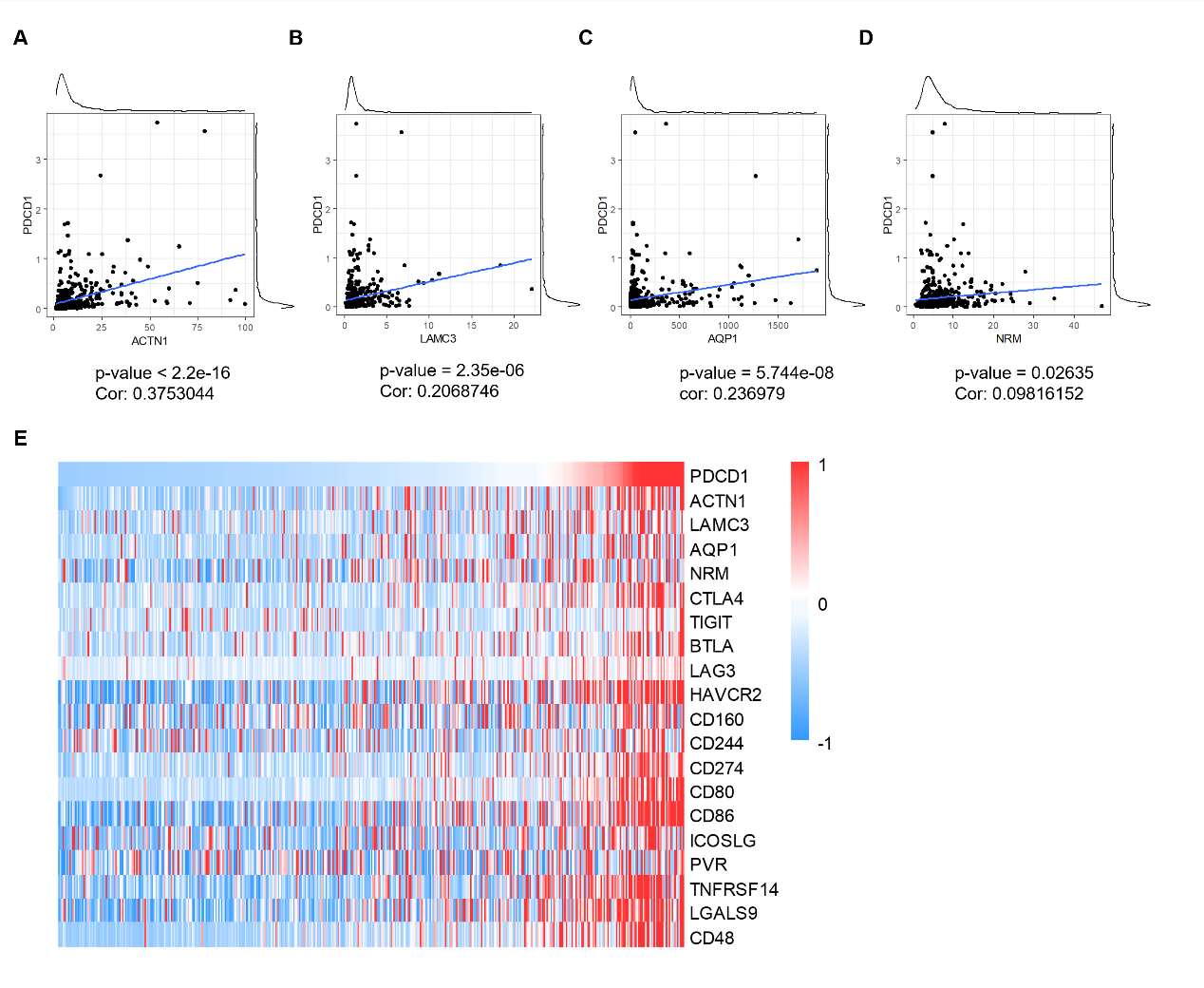


**Supplementary** **Figure 4. Correlation analysis: PDCD1 with ACTN1, LAMC3, AQP1, NRM, and exhaustion-related molecules in TCGA samples. A.** Correlation between PDCD1 and ACTN1. **B.** Correlation between PDCD1 and LAMC3. **C.** Correlation between PDCD1 and AQP1. **D.** Correlation between PDCD1 and NRM. **E**. Heatmap of the correlation between ACTN1, LAMC3, AQP1, and NRM and the expression of 39 exhaustion-related molecules, TCGA samples sorted by PDCD1 expression.

**Table S1. List of 132 EMT-associated genes related to grade II/III gliomas prognosis.** The expression profiles of 200 EMT-associated genes were extracted from TCGA LGG data. Univariate Cox regression analysis was performed using the coxph function in R to identify genes significantly associated with grade II/III gliomas prognosis (P < 0.05). This table lists the 132 EMT-associated genes that were found to be significantly related to prognosis in grade II/III gliomas patients.

**Table S2. Differentially expressed genes (DEGs) between C1 and C2 molecular subtypes.** Gene expression profiles of C1 and C2 molecular subtypes were compared using the limma package in R. Genes with |log2FC| > 1 and FDR < 0.01 were considered differentially expressed. A total of 1535 DEGs were identified, including 1291 up-regulated and 244 down-regulated genes in C1 compared to C2 subtypes. The table lists the DEGs along with their log2FC and FDR values.

**Table S3. Prognosis-related genes identified by univariate Cox regression analysis.** To identify genes associated with prognosis, univariate Cox regression analysis was performed on the survival data using a threshold of P < 0.01. A total of 874 prognosis-related genes were identified at this significance level. The table lists the genes along with their hazard ratios, confidence intervals and P values from the univariate Cox regression model.
